# Medicinal Plants for Viral Respiratory Diseases: A Systematic Review on Persian Medicine

**DOI:** 10.1155/2023/1928310

**Published:** 2023-02-10

**Authors:** Mahdie Hajimonfarednejad, Mohadeseh Ostovar, Fatemeh Sadat Hasheminasab, Mohammad Ali Shariati, Muthu Thiruvengadam, Mohammad Javad Raee, Mohammad Hashem Hashempur

**Affiliations:** ^1^Infertility Research Center, Shiraz University of Medical Sciences, Shiraz, Iran; ^2^Department of Persian Medicine, School of Medicine, Shiraz University of Medical Sciences, Shiraz, Iran; ^3^Pharmacology Research Center, Zahedan University of Medical Sciences, Zahedan, Iran; ^4^K. G. Razumovsky Moscow State University of Technologies and Management (The First Cossack University), 73 Zemlyanoy Val, Moscow 109004, Russia; ^5^Kazakh Research Institute of Processing and Food Industry, Semey Branch of the Institute, 238«G» Gagarin Ave., Almaty 050060, Kazakhstan; ^6^Department of Crop Science, College of Sanghuh Life Science, Konkuk University, Seoul 05029, Republic of Korea; ^7^Center for Nanotechnology in Drug Delivery, Shiraz University of Medical Sciences, Shiraz, Iran; ^8^Research Center for Traditional Medicine and History of Medicine, Department of Persian Medicine, School of Medicine, Shiraz University of Medical Sciences, Shiraz, Iran

## Abstract

**Introduction:**

Many medicinal plants have been introduced in Persian medicine references for various respiratory disorders. Considering the growing interest in herbal medicines, this review aimed to introduce medicinal herbs recommended by Persian Medicine (PM) references for respiratory diseases and to discuss their activity against respiratory viruses.

**Methods:**

The medicinal plants recommended for respiratory disorders were extracted from the main PM textbooks. Subsequently, their activity against respiratory viruses was systematically investigated via queries of scientific databases.

**Results:**

Searching PM references for medicinal plants used in the management of respiratory disorders yielded 45 results. Of them, 18 possess antiviral activity against respiratory viruses. There were 29 in vitro studies (including studies on human cell lines) and 5 in vivo studies.

**Conclusion:**

This research demonstrated that many of the medicinal plants mentioned for the respiratory diseases in PM have considerable activity against respiratory viruses. However, human studies regarding the reported medicinal plants are scarce.

## 1. Introduction

Viral respiratory infections are one of the most prevalent causes of medical consultations globally [1]. Known for a variety of clinical pictures, from self-limited upper respiratory tract diseases to life-threatening ones [2, 3], these infections deeply influence the quality of life and have a noticeable economic burden [4–6]. Additionally, the World Health Organization reports respiratory infections as the main reason for mortality among all infectious diseases [7]. Respiratory syncytial virus, influenza virus, metapneumovirus, parainfluenza viruses, adenoviruses, bocaviruses, rhinoviruses, and coronaviruses are respiratory viruses that are associated with epidemic or endemic infections in all continents [8]. Moreover, several viruses of the herpesvirus family, including cytomegalovirus, herpes simplex, varicella-zoster virus, human herpesvirus 6, and Epstein-Barr virus, may also be responsible for respiratory disease in immunocompromised individuals [8, 9]. The world is experiencing the third pandemic of severe acute respiratory syndrome coronavirus 2 (SARS-CoV-2) in the present century [10]. This novel coronavirus disease (COVID-19) is currently the most serious concern for the international community. It is a viral respiratory disease for which no effective treatment has yet been identified [11].

**Figure 1:**
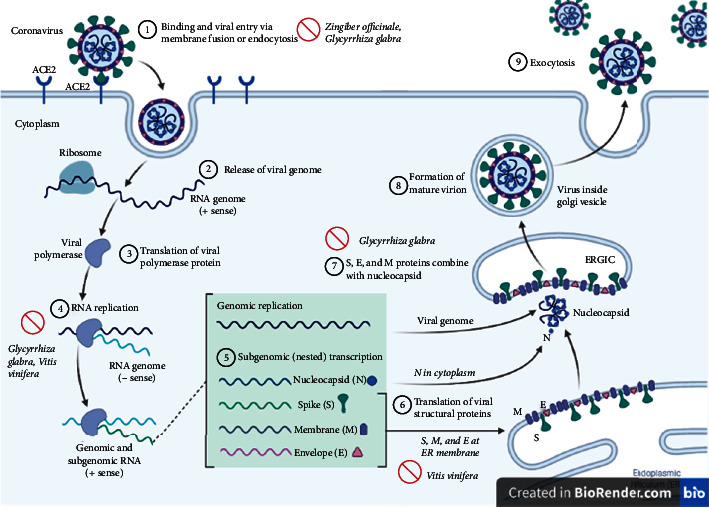
Plausible mechanisms for antiviral activity of medicinal plants, which may interrupt the coronavirus replication cycle.

Statistical analysis of studies related to the SARS 2002 outbreak indicated that the integration of traditional Chinese medicine (TCM) with conventional medicine could reduce morbidity and mortality rates as compared with mere conventional therapy [12]. Additionally, various traditional medical systems have brought up the issue of respiratory infections and related treatments [13–17]. Based on the humoral theory, Persian Medicine (PM) is an ancient medical system with multiple options for treating diseases and managing complications [18–21]. Specifically, numerous remedies have been reported in PM references for the treatment of various respiratory disorders, including asthma and pneumonia [22]. Additionally, there are several plausible mechanisms for the antiviral activity of these medicinal plants.  Figure 1 shows some of the proposed mechanisms, which may interrupt the coronavirus replication cycle [23–25]. Using herbs rather than contemporary drugs in COVID-19 therapy may have a wide variety of benefits and advantages, from a cheaper price and better worldwide availability to lesser adverse events, a better attitude of the general population towards them, and a decreased demand for conventional drugs and hospitalization [26–28].

Considering the global spread of viral respiratory infections, especially COVID-19, and the lack of any proven treatment in many cases, this research aimed to introduce medicinal plants recommended for respiratory diseases in PM and to review their activity against respiratory viruses according to current biomedical literature.

## 2. Methods

*Ketab al-Hawi fi al-Teb* (Continens) by Rhazes (9^th^ and 10^th^ centuries), *Qanun fi al-Ṭeb* (Canon of Medicine) by Avicenna (10^th^ and 11^th^ centuries), *Tebb-e Akbari* (Akbari's Medicine) by *Mohammad Akbar Arzani* (18^th^ century), *Exir-e Azam* (The Great Panacea) by *Nazem Jahan* (18^th^ and 19^th^ centuries), and *Makhzan al-Advieh* (Storehouse of Medicaments) by *Aghili Shirazi* (18^th^ century) are the most important and comprehensive textbooks of PM. They also comprise the references in the Ph.D. program for PM in Iran. Chapters related to respiratory disorders (respiration or *tanaffos*; lung or *shosh*, *riyah*; asthma or *rabv*; dyspnea with rapid and shallow breathing, cough or *sorfeh*, sputum or *nafth* in Persian) were selected and carefully searched for recommended medicinal plants.

The suggested medicinal plants were searched for their scientific and common names in English. Subsequently, medical English and Persian databases including MEDLINE, Scopus, Iranmedex, SID, Magiran, Web of Science, and Google Scholar were systematically searched. Each herb was searched along with keywords including “antivirus,” “coronavirus,” and “COVID-19.”

Two researchers independently screened the articles, reading their abstracts and titles to identify potentially eligible studies. Thereafter, full texts were obtained and read to determine the final included articles. In addition, the references of the retrieved articles were manually searched to identify other potentially eligible studies. Papers published in languages other than English or Persian were excluded. In addition, review articles and conference papers were not included in this systematic review. Moreover, research studies on nonrespiratory viruses and viruses not pathogenic for humans were excluded from the study. Any disagreement was resolved by discussion. The extracted data included plant scientific name, Persian name, English common name, used part of the herb, studied antiviral effect, and study type (including in-vitro, animal, and clinical). It should be noted that each plant's main compounds and route of traditional administration were added based on the PDR for herbal medicines (3^rd^ edition) [29] and *Makhzan al-Advieh* (Storehouse of Medicaments) by *Aghili Shirazi*, respectively.

## 3. Results

Forty-five medicinal plants recommended for respiratory diseases by PM resources were extracted in the first phase of the study. Overall, eighteen of the herbs recommended by PM resources for respiratory diseases have evidence regarding activity against viruses that can cause infectious respiratory disorders. Most of the research studies in this regard were in vitro studies. In addition, most of the mentioned plants were reported to act against influenza viruses (Table 1).

## 4. Discussion

The viral respiratory infection outbreaks promoted the conduct of studies with the purpose of evaluating novel medications, especially natural-based remedies, resulting in the discovery of potential drugs. The effectiveness of various herbs is published as the result of studies designed as case series, clinical trials, and systematic reviews [58–60]. These research studies encouraged further investigations to elucidate the potential of herbal compounds to manage coronavirus infections [61–63].

According to previous records regarding SARS 2002, TCM in combination with routine drugs has been far more effective than conventional therapy alone [12, 60, 61]. TCM physicians prescribed herbal remedies, which are known for their anti-inflammatory, antiviral, and immunomodulatory properties, for better management [12]. Studies have shown that these medicinal plants decrease the mean needed dosage of medications such as corticosteroids in severe cases and also diminish the adverse effects of some drugs. There are some reports that using corticosteroids for managing viral respiratory infections may lead to some adverse events (e.g., the development of fungal infection and femoral head necrosis). According to the results of 24 trials used in a meta-analysis, no long-term side effects due to taking high-dose corticosteroids were reported in integrative treatment (i.e., a combination of herbal drugs and conventional treatment) [61, 64].

It should be noted that some ancient medical systems, such as PM, TCM, and Unani medicine, have individualized approaches (phenotype-based personalized medicine) to treatment. Traditional practitioners consider gender, age, season, comorbidities, and many other patient characteristics to diagnose and manage different diseases [12, 65–67].

Human society is currently struggling with the COVID-19 pandemic, and no efficient drugs have been identified as of yet. Therefore, emphasis is placed on preventive measures and symptomatic therapies [68]. Regarding this, numerous research studies have been done to evaluate the safety and efficacy of preventive, therapeutic, supportive, or rehabilitative medicaments recommended in various traditional medical systems [15, 24, 69, 70]. The emphasis of the World Health Organization on the integration of traditional, complementary, and alternative medicine in the conventional health system and the growing interest in natural products for the management of diseases highlight the necessity of studies on different aspects of traditional medicines to reinforce the scientific evidence basis for natural remedies [19, 71].

Several research studies have previously been done to assess the efficacy of medicinal herbs reported in folklore or traditional medicine systems of different countries against various viral or bacterial respiratory infections [14]. A study in Guatemala assessed the antibacterial effect of 68 herbs traditionally applied for respiratory ailments. According to the results, 28 medicinal plants possessed inhibitory effects on one or more gram-positive bacteria, including *Streptococcus pneumonia*, *Staphylococcus aureus*, and *Streptococcus pyogenes* [72]. Another study investigated the antiviral properties of 44 Chinese herbs against the respiratory syncytial virus and demonstrated 27 medicinal plants with antiviral activity against this virus [73].

**Figure 2:**
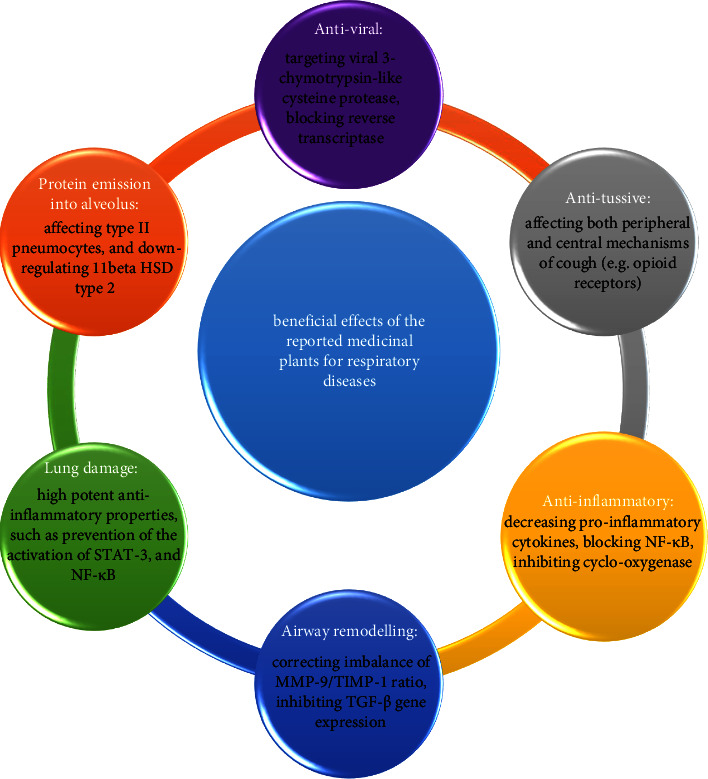
Beneficial effects of medicinal plants recommended by Persian medicine for respiratory disorders and some of their reported mechanisms.

The current study reviewed the antiviral properties of medicinal plants recommended for respiration disorders in PM. The antiviral effects of 18 (out of 45) herbs have been investigated and confirmed by experimental studies to date. Only some of the studies were performed on humans, while preclinical studies comprised the majority of the reports. The mentioned plants for respiratory disorders have antiviral activity as well. They have several other beneficial effects for patients with viral respiratory infections, including COVID-19 [23, 74–78] (Figure 2).

Among these research studies, the efficacy of *Glycyrrhiza glabra*, *Urtica dioica*, and *Nigella sativa* against coronaviruses has been confirmed [33]. These herbs not only possess antiviral activity but can also be used to alleviate symptoms associated with respiratory infections. For example, Glycyrrhizin, an active component (a triterpenoid saponin) of *Glycyrrhiza glabra* (licorice) root, has shown remarkable antiviral effects against coronavirus isolated from patients with SARS. Virus replication is inhibited when a nitrous oxide donor is added to the culture medium, and it was shown that glycyrrhizin induces nitrous oxide synthase in Vero cells. Also, glycyrrhizin lowers the expression of viral antigens and is able to inhibit the adsorption and penetration of the virus [33]. Moreover, this herb has exhibited antitussive activity on sulphur dioxide-induced cough in experimental rats [79]. In another study, rats with carrageenan-induced paw edema were treated with the hydroalcoholic extract of *Glycyrrhiza glabra* root. Its potent anti-inflammatory activity has been shown. This extract inhibited the migration of leukocytes dose-dependently, with anti-inflammatory effects comparable to indomethacin [80].

An in vitro study demonstrated that *Nigella sativa* extract has antiviral action by preventing coronavirus replication [57]. Thymoquinone, an important constituent of *Nigella sativa*, has been assessed for its antitussive property in guinea pigs. This constituent significantly subsided the cough induced by the nebulized solution of citric acid (20%). Additionally, pretreatment with naloxone leads to suppression of its antitussive effect, indicating stimulation of opium receptors as the mechanism [81]. Furthermore, analgesic and anti-inflammatory activities of the aqueous extract of *Nigella sativa* have been confirmed in rats via carrageenan-induced paw edema and hot plate reaction time, respectively [82].

Among herbs that have been recommended in PM references for respiratory disorders, the activity of 18 medicinal plants against respiratory viruses has been confirmed to date. Further studies are needed to evaluate whether other suggested medicinal plants have any effect against respiratory viruses or not. Further clinical studies should be considered a very important step towards the utilization of these plants in clinical practice. Also, further studies are necessary to compare the efficiency and safety of these herbs with conventional antiviral drugs. Another limitation of this research was the inclusion of only English and Persian papers.

## 5. Conclusion

Due to challenges with efficacy and safety, high costs, and limited worldwide availability of conventional treatments, the use of herbal medications for the management of viral respiratory infections is increasing. This systematic review showed antiviral activity (especially against influenza viruses and coronaviruses) for a significant portion of the medicinal herbs recommended for respiratory disorders in PM. However, not enough investigations have been conducted to confirm the efficacy of several of these plants on viral respiratory infections. Lack of or scant clinical studies is the main challenge in this regard; more vigorous research is suggested.

## Figures and Tables

**Table 1:** Medicinal plants recommended by Persian medicine for respiratory diseases with antiviral activity against respiratory viruses.

No	Scientific name	Persian name	English common name	Main compounds	Route of traditional administration	Used part or ingredient of the herb for biomedical research	Targeted virus	Studied cells/animals/populations	Results		
1	*Portulaca oleracea* L.	*Khorfeh*	Common purslane	Flavonoids alkaloids polysaccharides omega-3 fatty acids	Oral/fresh juice	Aqueous extract	Influenza a virus (IAV) (H1N1)	MDCK and A549 cell lines [30]	IAV infection was inhibited at the entry stage		
2	*Foeniculum vulgare* Mill.	*Raziyaneh*	Fennel	Transanethole fenchon estragole	Oral/decoction	Ethanol extract	Influenza a virus (H5N1)	MDCK cell line [31]	Plaque reduction (82.8%) in 300 *μ*g/ml of plant extract		
3	*Cydonia oblonga* Mill.	*Beh*	Quince	Cyanogenic glycosides: amygdalin mucilages fatty oil	Oral/paste	Its fruit extract was used for collecting 3-affeoylquinic acid	Influenza virus	Hemagglutination inhibition [32]	Its fruit extract significantly (*p* < 0.001) inactivated the virus		
4	*Glycyrrhiza glabra* L.	*Shirin-Bayan*	Licorice	Triterpene saponins glycyrrhetic acid flavonoids isoflavonoids glycyrol	Oral/decoction	Glycyrrhizin	Corona SARS virus	Vero cells [33]	It inhibited virus replication, adsorption, and penetration		
						Glycyrrhizin	Corona SARS virus	Vero cells from ATCC (ATCC CCL81) [29]	It inhibited SARS-CoV replication by changing the interaction pattern of virus to the cell receptor		
						Glycyrrhizin	Influenza virus	BALB/c mice (8 weeks) and MDCK cell [34]	It protected mice that exposed to a lethal load of virus. “*When mice infected with 20 and 10 LD50s of influenza virus were treated with it, 100 and 70% of the mice, respectively, survived over 21 days*.” in addition, it has no inhibitory effect on the virus replication (up to forty-eight hours post-infection) in the in-vitro study		
5	*Ziziphus jujuba* Mill.	*Annab*	Jujube	Triterpene saponins mucilage tannins	Oral/decoction	Betulinic acid	IAV (PR/8)	A549 cells and C57BL/6 mice (6 to 7 weeks of age) [35]	It showed strong antiviral activity against the virus (about 98%) at the concentration of 50 *μ*M and lesser activity against the virus (about 30%) at the concentration 10 *μ*M. Also, the animal study showed that betulinic acid significantly reduced the virus induced pulmonary pathology		
6	*Cucurbita pepo* L.	*Kadoo*	Pumpkin	Steroids fatty oil unusual amino acids	Oral/decoction	Oil extracted	Parainfluenza virus type-3	AGMK and MDBK cell lines [36]	It showed selective inhibitory effect against the virus		

7	*Cinnamomum camphora* (L.) J.Presl	*Kafoor*	Camphor	D-camphor linalol cineole	Nasal/inhalant Oral/decoction Topical/boiled	Camphecene (from ethanolamine and camphor)	IAV (H1N1)	MDCK cells (ATCC CCL 34) and BALB/c mice (aged 6 to 8 weeks) [35]	It decreased the number of virions fusing their envelopes with endosomal membranes. Camphecene significantly decreased the viral pathogenicity and attenuated the growth fitness in mice lung tissue		
						Camphor	IAV (H1N1)	MDCK cells [34]	Camphor blocked the viral ion channel M2. Then, it prevented the proton flow into the virions and its envelope fusion		
						Camphor	IAV (H5N1)	MDCK, and U-87 MG cells [37]	Inhibition of viral hemagglutinin in early stages of virus replication		
						Camphor derivative 1,7,7-trimethylbicyclo [2.2.1] heptan-2-ylidene-aminoetha 34nol (camphecene)	Influenza virus	MDCK cells (ATCC # CCL-34) and BALB/c mice (aged 6 to 8 weeks) [38]	Suppression of the virus replication it reduced the infectious titer of the virus in mice lung tissue		
8	*Vigna radiata* (L.) R.Wilczek	*Maash*	Mung bean	Phenolic acids flavoniods tannins	Oral/decoction	Its sprouts' methanol extract	Respiratory syncytial virus (RSV)	Vero and MRC-5 cell lines [39]	It induced IL-6, IL-1, IFN-*β*, and TNF-*α* in the studied cell lines		
9	*Cicer arietinum* L.	*Nokhod*	Chickpea	Proteins globulins fatty acids	Oral/decoction	Methanol extract	Parainfluenza-3 viruses	Madin-Darby bovine kidney and Vero cell lines [40]	Its extract had cytopathogenic inhibitory effect		
10	*Cinnamomum verum* J. Presl	*Darchin*	Cinnamon	Cinnamaldehyde weiterhin cinnamylacetate, cinnamyl alcohol tannins	Oral/decoction	Synthesized silver nanoparticles using cinnamon	Influenza virus (H7N3)	Vero cells [41]	It was effective against the viral infection. Its effectiveness increased when a pretreatment (before virus introduction to the cells) by it was added (compared with treatment only after infection)		
						Hot water extract of the plant	Human respiratory syncytial virus (HRSV)	Both human upper (HEp-2) and lower (A549) respiratory tract cell lines [42]	It dose-dependently inhibited HRSV-induced plaque formation in both cell lines. In addition its efficacy increased when given before infection. It inhibited “F protein production and syncytium formation to interfere with HRSV spreading”		
						Essential oils	H1N1 virus	MDCK cells (CCL-34, ATCC) [43]	The essential oil inactivated free-virus particles. It could interfere with virion envelope structures and its entry into the cells		
11	*Piper nigrum* L.	*Felfel siah*	Black pepper	Sabinene limonene caryophyllene betapinene	Oral/with honey or sugar	Combined methanol/dichloromethane extract of its fruits	Coxsackie virus type B3 (CVB3)	Vascular smooth muscle cells [44]	It had cytopathic inhibition effect		
						Chloroform and methanolic extracts	Human para influenza virus (HPIVS)	HeLa cell lines [45]	The extracts had inhibitory effect on the virus		
12	*Ficus carica* L.	*Anjir*	Fig	Furanocoumarins fruit acids mucilages pectin vitamin B and vitamin C	Oral/boiled with honey	Methanolic, hexanic, ethyl acetate, hexane-ethyl acetate, and chloroformic extracts of the fruit	Echovirus type 11 (ECV-11) and adenovirus (ADV)	ATCC CCL-81 (kidney cells of the African green monkey cercopithecus aethiops) [46]	The hexanic and hexane–ethyl acetate extracts inhibited viruses replication (at 78 mgmL^−1^.concentration)		

13	*Vitis vinifera* L.	*Maviz*	Common grape	Flavonoids anthocyanin vitamin A and vitamin B	Oral/decoction	Tea infusions from grape skins	Influenza virus	MDCK cells [47]	It protected MDCK cells against the virus (at 100 mg/ml concentration)		
						Aqueous, ethanol and acetone extracts of grape pomace	Influenza viruses (H5N1)	MDCK cells [48]	Its antiviral effects confirmed using plaque reduction assay		
14	*Zingiber officinale* Roscoe	*Zanjebil*	Ginger	Zingiberene arcurcumene, *β*-bisabolene	Oral/decoction, jam	Hot water extracts of fresh and dried ginger fruits	Human respiratory syncytial virus	Human upper (HEp-2) and lower (A549) respiratory tract cell lines [49]	The extract (from fresh fruits) inhibited the virus induced plaque formation in a dose-dependent manner in both cell lines
15	*Punica granatum* L.	*Anar*	Pomegranate	Tannins punicalin punicalagin	Oral/boiled with almond oil	Pomegranate polyphenol extract (PPE)	IAV (H3N2)	MDCK cells [50]	It inhibited the virus replication. In addition, “punicalagin blocked replication of the virus RNA, inhibited agglutination of chicken RBC's by the virus and had virucidal effects.”	
						Peels' ethyl alcohol extract	IAV	MDCK cells [51]	Its different extract types inhibited thew IAV	
						Peels' ethyl alcohol extract	IAV (H1N1; PR8)	MDCK cells [52]	It had inhibitory effects on the adsorption, polymerase activity, RNA replication, and protein expression of the virus	
						Peels' ethanol extract	Adenovirus	Hep-2 cell line [53]	The extract was effective against the virus (IC_50_ of 5.77 mg/mL)	
16	*Urtica dioica* L.	*Gazaneh*	Common nettle	Histamine serotonin acetylcholine formic acid	Oral/decoction	Urtica dioica agglutinin from its rhizomes	Respiratory syncytial virus (RSV) and IAV	HeLa and MDCK cells [54]	It inhibited the virus-induced cytopathicity	
						N-acetylglucosamine-specific stinging nettle lectin	Different SARS-CoV strains	BALB/c mice and Vero cells [55]	Its administration (5 mg/kg) significantly protected animals against a lethal infection. Regarding the in-vitro study, it had inhibitory effects on the virus replication, only if added before its adsorption	
17	*Mentha × piperita* L.	*Na'na*	Peppermint	Piperitone, *β*-caryophyllene, germacren D, 1,8-cineole, limonene, diosmin, hesperidin, quercitrin, thymonin, apigenine-7-glucuronide	Nasal/vapor bath oral/decoction	Ethanol extract from its leaves	Respiratory syncytial virus (RSV)	Hep-2 cell line [56]	It had a significant antiviral activity (IC_50_ of 10.41 lg/mL)	
18	*Nigella sativa* L.	*Shooniz*	Black seed	Nigellon, thymoquinone, thymohydroquinone, dithymoquinone, thymol, carvacrol, *α* and *β*-pinene, d-limonene, d-citronellol, p-cymene	Oral/decoction Topical/oil	Its seeds' ethanolic extract	Corona virus	HeLa-CEACAM1a (HeLa-epithelial carcinoembryonic antigen-related cell adhesion molecule 1a) cells [57]	Its administration had a significant effect on IL-8 level. In addition, it decreased the virus load	

Hep-2: human larynx epidermal carcinoma; MDCK: Madin-Darby canine kidney; AGMK: African green monkey kidney, MDBK: Madin-Darby bovine kidney.

## Data Availability

The data used to support the findings of this study are included within the article.
